# Functional evaluation outcomes correlate with histomorphometric changes in the rat sciatic nerve crush injury model: A comparison between sciatic functional index and kinematic analysis

**DOI:** 10.1371/journal.pone.0208985

**Published:** 2018-12-12

**Authors:** Tianshu Wang, Akira Ito, Tomoki Aoyama, Ryo Nakahara, Akihiro Nakahata, Xiang Ji, Jue Zhang, Hideki Kawai, Hiroshi Kuroki

**Affiliations:** 1 Department of Development and Rehabilitation of Motor Function, Human Health Sciences, Graduate School of Medicine, Kyoto University, Kyoto, Japan; 2 Department of Motor Function Analysis, Human Health Sciences, Graduate School of Medicine, Kyoto University, Kyoto, Japan; Szegedi Tudomanyegyetem, HUNGARY

## Abstract

Elucidating whether there is a correlation between biomechanical functions and histomorphometric data in the rat sciatic nerve crush injury model would contribute to an accurate evaluation of the regeneration state without sacrificing animals. The gold standard for functional evaluation is the sciatic functional index (SFI) despite there being intrinsic shortcomings. Kinematic analysis is considered a reliable and sensitive approach for functional evaluation, most commonly assessed as ankle angle at various phases of a gait cycle. Studies utilizing the toe angle for functional evaluation are scarce, and changes in the toe angle following surgery remain unknown. The present study assessed correlations of ankle angle, toe angle and SFI with histomorphometric data, aiming to determine which parameters most accurately reflect changes in histomorphometric data over time. Six Lewis rats were designated as the control group. 30 animals received surgery, six of them were randomly selected on the first, second, third, fourth, and sixth week after surgery for measurements of ankle and toe angles in the “toe-off” phase, and for evaluation of SFI. Histomorphometric analysis were also performed, to determine the number of myelinated nerve fibers, diameters of myelinated nerve fibers, axon diameters, and myelin sheath thicknesses. Furthermore, we investigated changes in ankle angle, toe angle, SFI, and histomorphometric data over time, as well as correlations between ankle angle, toe angle, and SFI with histomorphometric data. The results revealed that changes in SFI, ankle angle, and toe angle highly correlate with histomorphometric data in the rat sciatic nerve crush injury model. Toe angle reflected changes in histomorphometric data with time more precisely than ankle angle or SFI did, and ankle angle was a better prognostic parameter than SFI.

## Introduction

The sciatic nerve is likely to undergo varying extent of crush injury in situations such as the fracture of pelvis, swelling resulting from muscle strains, or even prolonged sitting or lying with pressure on the buttocks [1]. Nevertheless, the outcome of a crush injury can be improved by axonal regeneration and re-myelination in humans and rodents, due to intrinsic regenerative abilities of a peripheral nerve. To achieve full recovery, the sciatic nerve generally undergoes three main processes after crush injury: Wallerian degeneration, axonal regeneration, and end-organ reinnervation[2]. Wallerian degeneration occurs within the first week after injury; the required time for axonal regeneration and end-organ reinnervation primarily depends on the distance from the distal stump to the organ. Functional recovery begins when regenerated axons reach the end-organs. Function gradually improves with nerve regeneration maturation process, including remyelination and axon enlargement, with sufficient maturation resulting in complete functional recovery [2]. The rat sciatic nerve crush injury model is commonly used to conduct studies of peripheral nerve injuries because rats are inexpensive to house and have similar nerve trunk distributions to humans [2]. Evaluations of repair processes are typically performed using functional measurements and histomorphometric analysis. Histomorphometric analysis rely on target tissue extraction from experimental animals, which is a terminal procedure, requiring animal sacrifice. Thus, identification of a correlation between function and histomorphometric data would be useful for evaluating the degree of sciatic nerve regeneration accurately while also allowing for reductions in the number of experimental animals that are sacrificed. Previous studies suggested a lack of correlation between functional evaluation and histomorphometric data [3],[4]. However, recent research provided evidence to the contrary, with findings indicating support a reliable correlation between function and histomorphometric data [5–7].

The sciatic functional index (SFI) is the gold standard for evaluating sciatic nerve function [8], and was first introduced by de Medinaceli et al. [9] in 1982 and have modified by Bain et al. [10] in 1989. Furthermore the SFI has been widely adopted by various functional evaluation studies of rat sciatic nerve injury [11–15]. However, there are several problems with the SFI, including automutilation, [16] joint contracture, and smearing of the print [17]; these problems can seriously affect its prognostic value. Additionally, some studies have demonstrated that the SFI is not reliable during the early stages of self-repair following sciatic nerve injury [18], and that it correlates poorly with other evaluation measures [8].

Kinematic analysis includes gait and walking analysis by tracking markers attached to relative bony landmarks or joints, and is being increasingly used. Devoid of the shortcomings attributed to SFI [19], [20], kinematic analysis is also recognized as a reliable and sensitive method of functional evaluation [21]. Although rats must be trained to walk on a treadmill, which is time-consuming, directly and clearly observing sciatic function by measuring the degree of a designated angle is a prominent advantage. Previous studies have focused on ankle angle determination during various phases of a gait cycle [22], [23]. Yurie et al. have found that the increment of toe angle during the terminal swing phase was accompanied by regeneration of the sciatic nerve after injury [24] but did not examine the change in toe angle over time. Studies evaluating toe angle remain very limited.

Several histomorphometric techniques are available to examine sciatic nerve crush injury. For example, methods include calculating numbers of myelinated nerve fibers by transverse sections with toluidine blue staining, Masson’s trichrome staining, or hematoxylin and eosin staining [25], as well as observing and calculating relevant parameters of the nerve fiber under a transmission electron microscope [26] or examining the length of regenerated sciatic nerve on longitudinal sections with fluorescent staining[14].

The aim of the present study was to investigate whether there is a correlation between histomorphometric changes and functional outcomes obtained by kinematic analysis or SFI, and we also would like to determine which functional evaluation reflects to changes best in histomorphometric data over time.

## Materials and methods

### Animals

A total of 36, 12-week-old male Lewis rats (250–300 g), were randomly assigned to two groups: the control group (n = 6) and the experimental group which received surgery (n = 30). Six rats were randomly selected from the experimental group at the first, second, third, fourth, and sixth weeks after surgery, thereby creating five sub-groups (1w, 2w, 3w, 4w, and 6w) for kinematic analysis, SFI evaluation, and histomorphometric analysis. The study was approved by the animal experimentation committee of Kyoto University, and all experiments were performed in accordance with the Guidelines of the Animal Experimentation Committee, Kyoto University (approval number: MedKyo17029).

### Surgery

Rats received general anesthesia by intraperitoneal injection of 64.8 mg/kg pentobarbital sodium and the surgical field was shaved. The left sciatic nerve was exposed with a lateral longitudinal straight incision from the greater trochanter to mid-thigh and then by blunt dissection between the quadriceps femoris and biceps femoris. When the sciatic nerve was exposed and detached from the surrounding tissues, a 2-mm-long crush injury was created at the site directly below the gluteal tuberosity, using a standard surgical hemostat. The injury site was marked using a stereomicroscope (Leica, Heidelberg, Germany) with a single 9–0 nylon (BEAR Medical Inc., Japan) epineural stitch at the proximal end of the injury, for later identification. The muscle and skin were then closed with 4–0 nylon sutures and rats were returned to cages.

### Kinematic analysis

**Fig 1:**
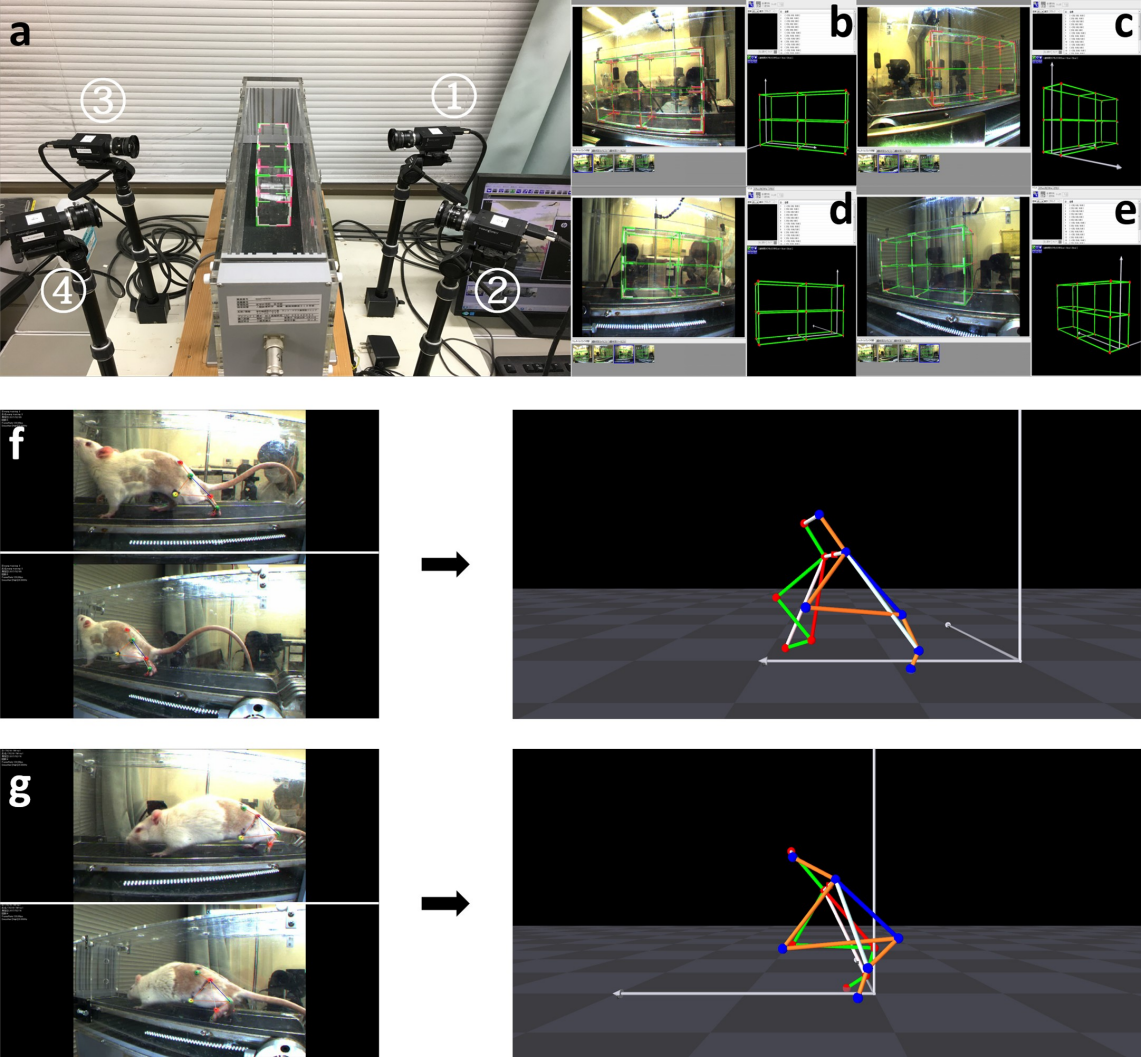
Panel a shows the treadmill with a 3-dimensional (3D) motion capture apparatus. The 3D motion capture apparatus is composed of four 120 Hz charged coupled device cameras and a set of software components used to process data. Two cameras were placed in line on the right and left sides of the treadmill. A custom calibration box was placed on the treadmill for subsequent calibration. Panels b-e show photos of the calibration box taken by cameras ➀, ➁, ➂, and ➃, respectively. By correspondingly marking red points of the 3D model of the calibration box on these photos, automatic calibration can occur. Panels f and g demonstrate that a 3D model of a rat’s left posterior limbs is built by synthesizes two photos taken by cameras on the left side. Images of rats in panels f and g were taken before surgery and at the first week after surgery, respectively.

In order to conduct kinematic analysis, a treadmill with a 3-dimensional (3D) motion capture apparatus (Kinema Tracer System, Kissei Comtec, Nagano, Japan) was used [27](Fig 1A). Before walking on the treadmill, all rats were anesthetized and equipped with markers according to a previous procedure [24]. Colored hemispheric markers were bilaterally attached to five key landmarks on the shaved skin, including the anterior superior iliac spine, greater trochanter, knee joint, lateral malleolus, and the fifth metatarsophalangeal joint. The tip of the fourth toe was marked with pink ink (paint marker, Nippon Paint Co., Ltd, Tokyo, Japan). The colors of the adjacent markers differed. Subsequently, in order to let the 3D motion capture apparatus correctly recognize various kinematic parameters of rats and reduce the effects of the fish-eye distortion caused by the camera lenses, a calibration process was conducted automatically using a calibration box (Fig 1B–1E). Following the calibration procedure, rats walked on a treadmill at speeds of 12 m/min and their posterior limb motions were recorded with cameras [27]. For subsequent analysis, 10 consecutive steps were recorded from each rat and the affiliated software automatically built 3D kinematic models based on tracing markers attached to the six above-mentioned landmarks (Fig 1G–1F). In the present study, two parameters were analyzed: (1) ankle angle in the “toe-off” phase, which was the angle formed by a line connecting the knee joint and lateral malleolus, and another line connecting the lateral malleolus and the fifth metatarsophalangeal joint; and (2) toe angle in the “toe-off” phase, which was the angle formed by a line connecting the fifth metatarsophalangeal joint and the tip of the fourth toe, and another extension line from the lateral malleolus to the fifth metatarsophalangeal joint. The degree of toe angle at plantarflexion is denoted as minus and that at dorsiflexion is denoted as plus. Averages of the two parameters were comprised the mean ankle angle and mean toe angle.

### SFI

A wooden walking alley (9 × 10 × 60 cm), bilaterally walled by plastic plates was prepared in advance, containing a darkened carton box at the end. The floor of the alley was covered with a paper tape (8.6 × 59.4 cm) and a brush was used to smear ink completely over the planta pedis of the rat. The rat was placed at the entrance of the alley and always walked straight through the alley into the carton box, leaving footprints on the paper tape. Three legible footprints were selected from the several footprints of each rat and three different parameters were measured: (1) distance from the heel to the third toe (print length; PL); (2) distance from the first toe to the fifth toe (toe spread; TS); and (3) distance from the second toe to the fourth toe (intermediate toe spread; ITS). The SFI was calculated according to the following formula:

SFI=‑38.3((EPL−NPL)/NPL)+109.5((ETS−NTS)/NTS)+13.3((EITS−NITS)/NITS)‑8.8

where E is the experimental side and N is the normal side. A value of 0 indicates normal function and a value of -100 indicates total impairment. The mean value of SFI was calculated from these three footprints.

### Histomorphometric analysis

Rats were administered overdosage (intraperitoneal injection of 129.6 mg/kg pentobarbital sodium) for euthanasia. A 5-mm-long sciatic nerve sample was dissected from the epineural stitch of each rat and immersed in a fixative containing 1.44% paraformaldehyde and 1% glutaraldehyde in 0.036 M phosphate buffer (pH = 6.8) at 4°C overnight, then washed with 0.1 M phosphate buffer (PB) for 20 min three times, and fixed with 1% osmium tetroxide in 0.1 M PB for 120 min. The sample was then dehydrated through 50%, 60%, 70%, 80%, 90%, and 99% ethanol aqueous solutions in sequence, each for 20 min, and finally in 100% ethanol for 30 min twice. Following this, the sample was penetrated with propylene oxide for 60 min twice, and through a mixed solution of propylene oxide (50%) and epon (Luveak; Nacalai Teque, Kyoto, Japan) (50%) and propylene oxide and epon (1:4) each for 90 min, then with epon only for 12 h, and polymerized with epon at 60°C overnight. Subsequently, 1-μm-thick transverse sections were prepared and examined under a light microscope (Nikon ECLIPSE 80i, Tokyo, Japan) after being stained with 0.5% (w/v) toluidine blue solution. A photo of a sciatic nerve transection at 200x magnification was taken, and the number of myelinated nerve fibers in three different random areas of 90,000 μm^2^ was calculated using ImageJ software (National Institutes of Health, Bethesda, MD, USA), accounting for at least 30% of the total area of the photo. The mean number of myelinated nerve fibers in three of the above-mentioned areas represented the number of myelinated nerve fibers. Moreover, the mean number of myelinated nerve fibers in a 90,000 μm^2^ area was adapted to the number of myelinated nerve fibers in a 10,000 μm^2^ area in order to more clearly express the results. Ultrathin transverse sections of the same tissue stained with uranyl acetate and lead citrate were also examined under a transmission electron microscope (Model H-7000; Hitachi High-Technologies, Tokyo, Japan). Ten pictures representing different random areas of the section were obtained at 2000x magnification, and the shortest diameter of myelinated nerve fiber (α), as well as the axon diameter (β), of each myelinated nerve fiber were measured. The myelin sheath thickness (γ) of each myelinated nerve fiber was calculated according to the following formula: (α-β)/2. Averages of these parameters (diameter of myelinated nerve fiber, axon diameter, and myelin sheath thickness) obtained from all myelinated nerve fibers were considered the mean diameter of myelinated nerve fiber, mean axon diameter, and mean thickness of myelin sheath, respectively.

### Statistical analysis

Data are presented as mean ± SEM. Differences were assessed for significance using one-way ANOVA. The Dunnett test was used to assess the significance of differences between the experimental groups and the control group. Tukey’s HSD test was applied to comparisons between the groups after surgery. We primarily focused on the comparisons of adjacent groups in this study because changes in these data demonstrated improvements under most circumstances, and only the change in SFI did not suggest a trend toward improvements from 1w to 2w. The correlation coefficients between kinematic analysis parameters and SFI with histomorphometric data were calculated using Spearman’s rho correlation analysis. The Meng-Rosenthal-Rubin method was used to compare correlation coefficients. Statistical significance was indicated by p <0.05.

## Results

### Kinematic analysis

**Fig 2:**
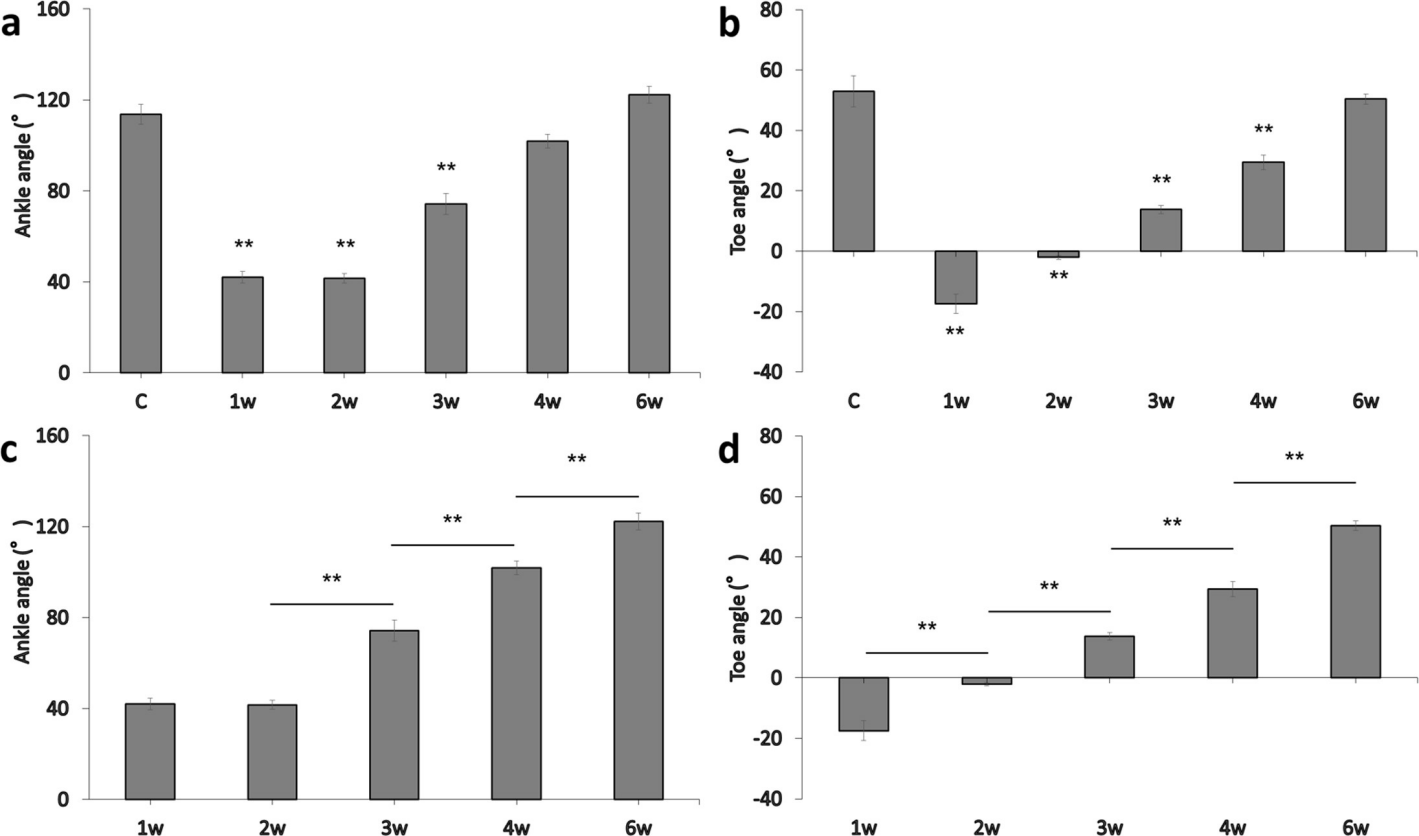
Panels a and b show changes in the ankle angle and toe angle, respectively, over time (error bars = standard error of the mean, SEM; **p < 0.01, compared to the control group). Panels c and d show ankle angle and toe angle, respectively, at different time points after surgery (error bars = SEM, **p < 0.01, comparison to adjacent groups).

Ankle angle generally improved upward after surgery but did not significantly improve from 1w to 2w (Fig 2). The ankle angle in the control group was 113.71 ± 4.40°, compared to 42.02 ± 2.54° at the first week, 41.65 ± 2.03° at the second week, 74.30 ± 4.58° at the third week, 101.89 ± 3.02° at the fourth week, and 122.26 ± 3.72° at the sixth week after surgery (Fig 2A). The 1w, 2w, and 3w groups differed significantly compared to the control group (p < 0.01), and comparisons of adjacent groups indicated significant differences between 2w and 3w, 3w and 4w, and 4w and 6w (p < 0.01) (Fig 2C).

Toe angle improved in an upward direction following surgery. The toe angle in the control group was 52.97 ± 5.14°, compared to -17.45 ± 3.23° in the first week, -2.03 ± 0.56° in the second week, 13.77 ± 1.28° in the third week, 29.41 ± 2.46° in the fourth week, and 50.39 ± 1.62° in the sixth week after surgery (Fig 2B). The 1w, 2w, 3w, and 4w groups were significantly different compared to the control group (p < 0.01), and comparisons of adjacent groups indicated significant differences between all adjacent groups following surgery (p < 0.01; Fig 2D).

### SFI

**Fig 3:**
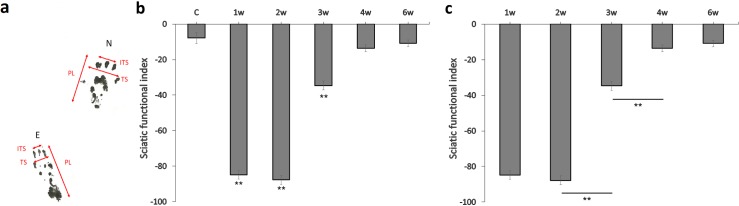
Panel a shows a pair of rat footprints one week following surgery. PL = print length, TS = toe spread, ITS = intermediate toe spread. Experimental side (E) is left and normal side (N) is right. Panel b shows changes in the sciatic functional index (SFI) over time (error bars = standard error of the mean, SEM; **p < 0.01, compared to the control group). Panel c shows the SFI at different time points after surgery (error bars = SEM, **p < 0.01, comparisons between adjacent groups).

SFI generally improved upward, although there was no trend toward improvement from 1w to 2w (Fig 3). The SFI value in the control group was -7.88 ± 3.10 (Fig 3B). Over time, the SFI values were -84.89 ± 2.38 in the first week, -87.76 ± 2.52 in the second week, -34.67 ± 2.54 in the third week, -13.66 ± 1.93 in the fourth week, and -10.92 ± 1.84 in the sixth week. The 1w, 2w, and 3w groups were significantly different compared to the control group (p < 0.01), and comparisons of adjacent groups indicated significant differences between 2w and 3w (p < 0.01) and between 3w and 4w (p < 0.01; Fig 3C).

### Histomorphometric analysis

**Fig 4:**
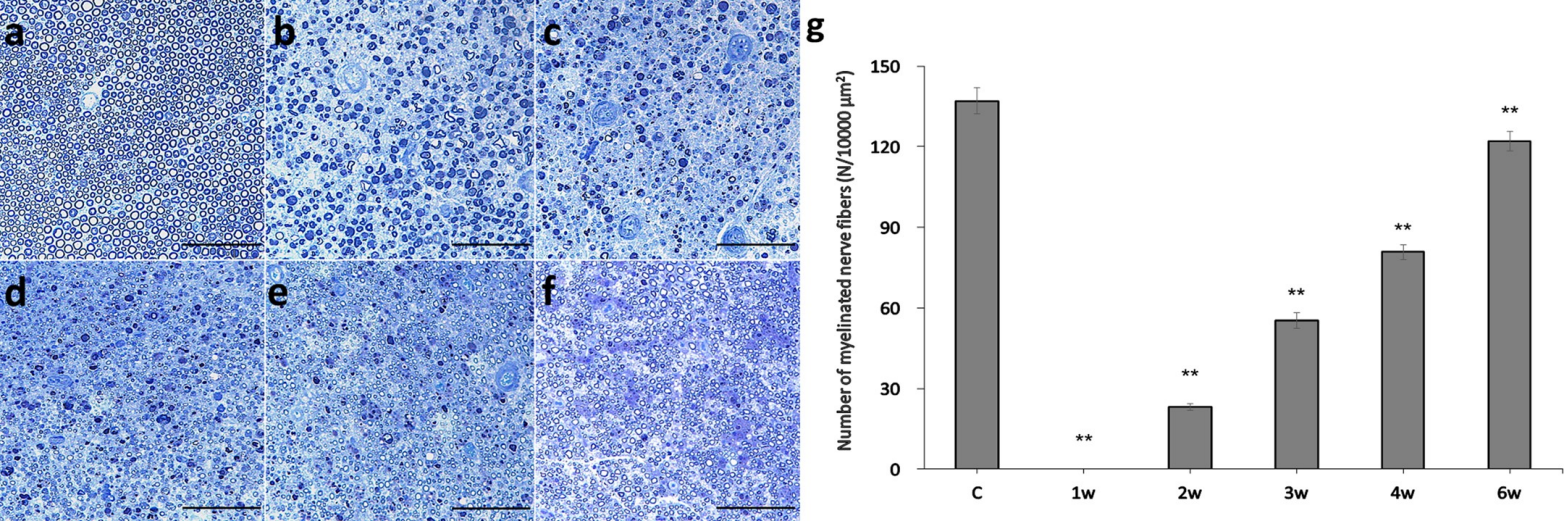
Panels a-f show transverse sections of myelinated nerve fibers stained with toluidine blue from the control group (a), as well as 1w (b), 2w (c), 3w (d), 4w (e) and 6w (f) groups (scale bars = 100 μm). Panel g shows a change in the mean number of myelinated nerve fibers in a 10,000 μm^2^ area over time (error bars = standard error of the mean, **p < 0.01, compared to the control group).

**Fig 5:**
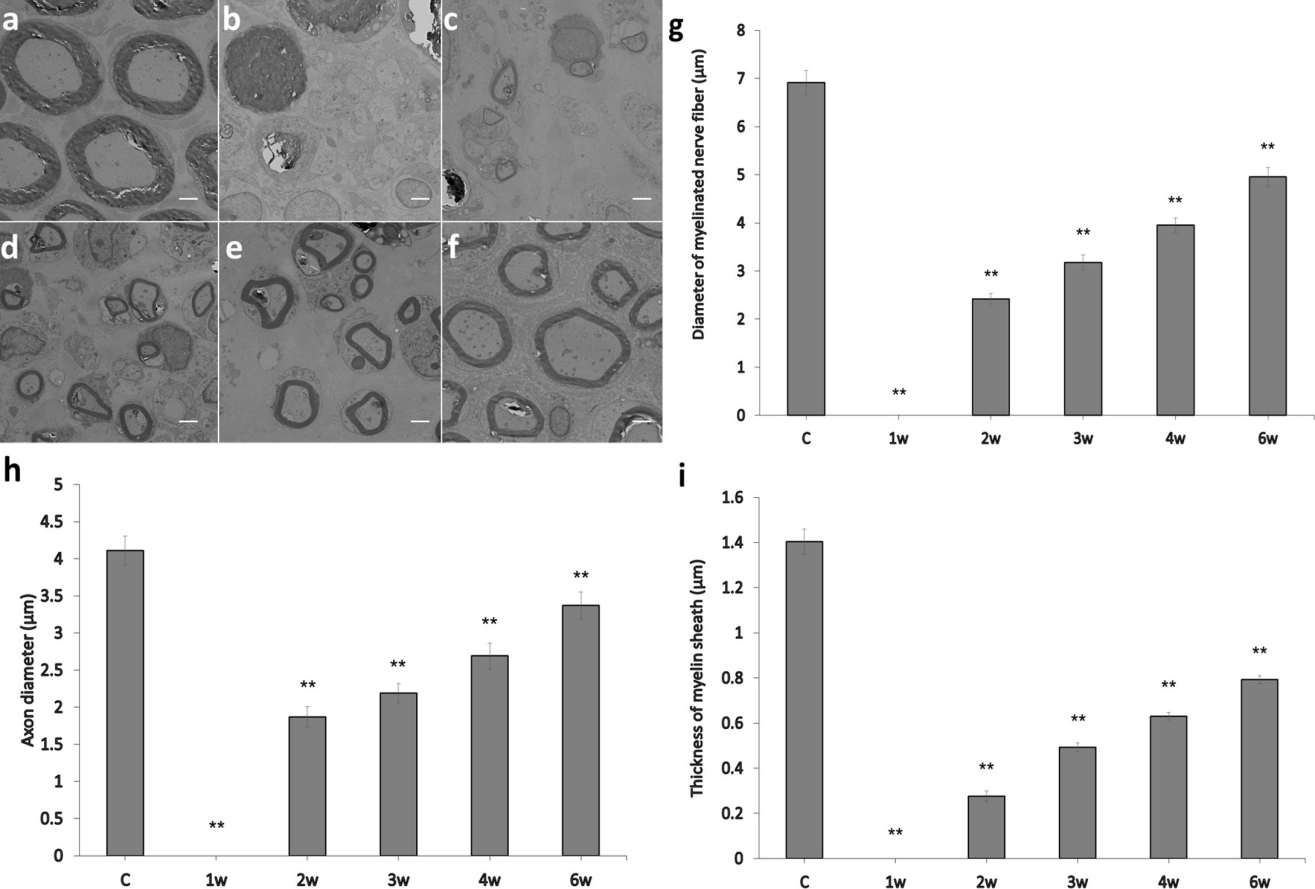
Panels a-f show transverse sections of myelinated nerve fibers using a transmission electron microscope, for the control group (a), as well as 1w (b), 2w (c), 3w (d), 4w (e) and 6w (f) groups (scale bars = 2 μm). Panels g, h, and i show changes in the diameter of myelinated nerve fibers, axon diameter, and myelin sheath thickness, respectively, over time (error bars = standard error of the mean, **p < 0.01, compared to the control group).

The number of myelinated nerve fibers, diameter of myelinated nerve fibers, axon diameter, and thickness of the myelin sheath all improved following surgery (Figs 4 and 5). Fig 4 shows that the number of myelinated nerve fibers was 137 ± 5 in the control group and changed from 0 in the first week following surgery to 23 ± 1 in the second week, 55 ± 3 in the third week, 81 ± 3 in the fourth week, and 122 ± 4 in the sixth week after surgery (Fig 4G). All other groups were significantly different compared to the control group (p < 0.01).

The diameters of myelinated nerve fibers in the control group and the 1w, 2w, 3w, 4w, and 6w groups were 6.92 ± 0.25 μm, 0 μm, 2.42 ± 0.11 μm, 3.17 ± 0.16 μm, 3.95 ± 0.15 μm, and 4.96 ± 0.19 μm, respectively (Fig 5G); the axon diameters were 4.11 ± 0.19 μm, 0 μm, 1.87 ± 0.14 μm, 2.19 ± 0.13 μm, 2.69 ± 0.17 μm, and 3.37 ± 0.18 μm, respectively (Fig 5H); the myelin sheath thicknesses were 1.40 ± 0.06 μm, 0 μm, 0.28 ± 0.02 μm, 0.49 ± 0.02 μm, 0.63 ± 0.02 μm, and 0.79 ± 0.02 μm, respectively (Fig 5I). The 1w, 2w, 3w, 4w, and 6w groups were significantly different compared to the control group in all three parameters (p < 0.01) (Fig 5G–5I).

### Correlation analysis

Table 1 shows significant correlations between each of ankle angle, toe angle, and SFI, with histomorphometric data including number of myelinated nerve fibers, diameter of myelinated nerve fibers, axon diameter, and myelin sheath thickness (p < 0.01); correlation coefficients for toe angle were always the highest and correlation coefficients for SFI were always the lowest. Moreover, the correlation coefficients for toe angle and the number of myelinated nerve fibers, diameter of myelinated nerve fibers, and axon diameter were always significantly higher than the correlations between ankle angle or SFI and histomorphometric data (Table 1).

**Table 1:** Correlations between sciatic functional index (SFI), ankle angle, and toe angle with the number of myelinated nerve fibers, diameter of myelinated nerve fibers, axon diameter, and myelin sheath thickness.

	SFI	Ankle angle	Toe angle
Number of myelinated nerve fibers	0.88	0.90	0.96**
Diameter of myelinated nerve fibers	0.86	0.87	0.93*
Axon diameter	0.82	0.84	0.91*
Myelin sheath thickness	0.87	0.89	0.93

*p < 0.05

**p < 0.01

When correlations with the number of myelinated nerve fibers, the diameter of myelinated nerve fibers, and axon diameter were evaluated, correlation coefficients for toe angle were always significantly higher than the correlation coefficients for ankle angle or SFI (*p < 0.05, **p < 0.01).

## Discussion

In this study, we aimed to determine whether correlations exist between ankle angle, toe angle, and SFI, and histomorphometric data. We also aimed to identify which of these parameters reflects temporary changes in histomorphometric data most accurately. Munro found no correlation between functional evaluations and histomorphometric analysis [3]. In contrast, Dellon and Mackinnonhad reported correlations between electrophysiology and the nerve fiber diameters or the number of nerve fibers, as well as between the peroneal functional index and nerve fiber diameter [28]. Therefore, the discrepancy regarding correlations between function and histomorphometric data necessitate further research. In the present study, functional evaluation consisted of both SFI and kinematic analysis, and histomorphometric analysis included four parameters, including the number of myelinated nerve fibers, the diameter of myelinated nerve fibers, axon diameter, and myelin sheath thickness. Kinematic analysis included measurements of the ankle angle and toe angle in the “toe-off” phase. We observed that all parameters (ankle angle, toe angle, and SFI) strongly correlated with the histomorphometric data.

The SFI demonstrated a strong correlation with histomorphometric data, although several problems remained. In the present study, print smearing did not interfere with measurement or calculation of the SFI. However, the foot drop caused by paralysis after surgery decreased measurement precision 2 weeks after surgery. A previous study also noted that foot drop and clawing of toes prevented animals from standing on their paws and interfered with obtaining a reliable paw print [18]. With respect to SFI measurements at the fourth and sixth weeks after surgery, we favor the explanation that the spontaneous repair process after sciatic nerve crush injury was very rapid, thus enabling the rats to stand on their paws and fully extend their toes, following reinnervation at the fourth week after surgery. There was no further improvement in toe extension (and no significant change in the SFI) at the sixth week after surgery, although functional improvement and nerve regeneration were progressing continuously and slowly.

Kinematic analysis provides an innovative option for functional evaluation. The analysis described in this study is not identical to the general video walking or gait analysis. Two cameras positioned from different directions recorded the posterior limb motions for one (left or right) side, similar to human eyes. Subsequently, using computer graphics, a 3D special digital model of the posterior limb could be constructed, and the ankle angle and toe angle were calculated by recapitulating the authentic dimensions closely. We assessed ankle angle and toe angle in the “toe-off” phase. By performing comparisons between the experimental groups, we found that the ankle angle and toe angle demonstrated clear changes in the “toe-off” phase, and initial kinematic outcomes were also strongly correlated with histomorphometric data. Moreover, the ankle angle in the “toe-off” phase reflects the recovery process following sciatic nerve injury. Specifically, the ankle angle was maximal in the “toe-off” phase of a step cycle period, meaning that ankle angle measurements in that phase were more reliable and sensitive than in any other cycle phase after sciatic nerve injury [22]. Although few previous studies considered toe angle as a meaningful parameter when conducting kinematic analysis, we found that the toe angle had similar characteristics to ankle angle; toe angle in the “toe-off” phase also reached the maximum in a step cycle period and accurately reflected the condition of the sciatic nerve because relevant foot muscles are innervated by the tibial nerve, the thickest main branch derived from the sciatic nerve.

Our results demonstrate upward improvement of both ankle and toe angles, and are consistent with changes in histomorphometric data including the number of myelinated nerve fibers, diameter of myelinated nerve fibers, axon diameter, and myelin sheath thickness after surgery, although ankle angle did not improve in the first two postoperative weeks. Importantly, while the ankle angle improved significantly between 2w and 6w, the toe angle improved significantly from 1w to 6w. Toe angle was also most strongly correlated with histomorphometric data, except for myelin sheath thickness. In addition, when compared to the control group, there were significant differences until 4w for toe angle values, which only occurred until 3w for ankle angle and SFI. In contrast, SFI did not show a trend toward improvement in the first two postoperative weeks, and there was minimal improvement from 4w to 6w. In summary, ankle angle and toe angle were both stronger indicators than SFI in this study, and the kinematic analysis was useful for functional evaluation following surgery, which is consistent with our original hypothesis.

Despite the clear benefits of kinematic analysis, the disadvantages of a complicated marker-attaching process and time-consuming data collection procedures cannot be overlooked. The SFI is simple to perform and is optimally applicable to the rat sciatic nerve crush injury model, despite poor performance in the rat sciatic nerve autograft or conduit model, due to its low sensitivity and intrinsic shortcomings [16], [17].

In conclusion, SFI, ankle angle, and toe angle strongly correlated with histomorphometric data in a rat sciatic nerve crush injury model (correlation coefficients >0.7). However, ankle angle and toe angle measurements more accurately reflecting histomorphometric data compared to the SFI in the rat sciatic nerve crush injury recovery model, and toe angle was a better predictor than ankle angle. Our consideration of toe angle as an informative parameter observed in kinematic analysis is innovative, and the results demonstrate both improvements in relation to functional evaluation, as well as greater sensitivity compared to ankle angle. Further studies are needed in order to determine the mechanistic basis for toe angle as a more sensitive measurement than ankle angle in the “toe-off” phase of early stage nerve regeneration. The present study supports that kinematic analysis is a more reliable and sensitive measurement, which could be applied to experiments in rats. Increased use of kinematic analysis will contribute to understanding of both nerve function and regeneration on a histological level during recovery following sciatic nerve injury.

## Supporting information

S1 Table Data for support information. (XLSX)
